# The mesolimbic system and the loss of higher order network features in schizophrenia when learning without reward

**DOI:** 10.3389/fpsyt.2024.1337882

**Published:** 2024-09-03

**Authors:** Elizabeth Martin, Asadur Chowdury, John Kopchick, Patricia Thomas, Dalal Khatib, Usha Rajan, Caroline Zajac-Benitez, Luay Haddad, Alireza Amirsadri, Alfred J. Robison, Katherine N. Thakkar, Jeffrey A. Stanley, Vaibhav A. Diwadkar

**Affiliations:** ^1^ Department of Psychiatry & Behavioral Neurosciences, Wayne State University School of Medicine, Detroit, MI, United States; ^2^ Department of Psychiatry, University of Texas Austin, Austin, TX, United States; ^3^ Department of Neurosurgery, University of Michigan, Ann Arbor, MI, United States; ^4^ Department of Physiology, Michigan State University, East Lansing, MI, United States; ^5^ Department of Psychology, Michigan State University, East Lansing, MI, United States

**Keywords:** schizophrenia, learning, memory, functional MRI, cognition, mesolimbic system

## Abstract

**Introduction:**

Schizophrenia is characterized by a loss of network features between cognition and reward sub-circuits (notably involving the mesolimbic system), and this loss may explain deficits in learning and cognition. Learning in schizophrenia has typically been studied with tasks that include reward related contingencies, but recent theoretical models have argued that a loss of network features should be seen even when learning without reward. We tested this model using a learning paradigm that required participants to learn without reward or feedback. We used a novel method for capturing higher order network features, to demonstrate that the mesolimbic system is heavily implicated in the loss of network features in schizophrenia, even when learning without reward.

**Methods:**

fMRI data (Siemens Verio 3T) were acquired in a group of schizophrenia patients and controls (n=78; 46 SCZ, 18 ≤ Age ≤ 50) while participants engaged in associative learning without reward-related contingencies. The task was divided into task-active conditions for encoding (of associations) and cued-retrieval (where the cue was to be used to retrieve the associated memoranda). No feedback was provided during retrieval. From the fMRI time series data, network features were defined as follows: First, for each condition of the task, we estimated 2^nd^ order undirected functional connectivity for each participant (uFC, based on zero lag correlations between all pairs of regions). These conventional 2^nd^ order features represent the task/condition evoked synchronization of activity between pairs of brain regions. Next, in each of the patient and control groups, the statistical relationship between all possible pairs of 2^nd^ order features were computed. These higher order features represent the consistency between all possible pairs of 2^nd^ order features in that group and embed within them the contributions of individual regions to such group structure.

**Results:**

From the identified inter-group differences (SCZ ≠ HC) in higher order features, we quantified the respective contributions of individual brain regions. Two principal effects emerged: 1) SCZ were characterized by a massive loss of higher order features during multiple task conditions (encoding and retrieval of associations). 2) Nodes in the mesolimbic system were over-represented in the loss of higher order features in SCZ, and notably so during retrieval.

**Discussion:**

Our analytical goals were linked to a recent circuit-based integrative model which argued that synergy between learning and reward circuits is lost in schizophrenia. The model’s notable prediction was that such a loss would be observed even when patients learned without reward. Our results provide substantial support for these predictions where we observed a loss of network features between the brain’s sub-circuits for a) learning (including the hippocampus and prefrontal cortex) and b) reward processing (specifically constituents of the mesolimbic system that included the ventral tegmental area and the nucleus accumbens. Our findings motivate a renewed appraisal of the relationship between reward and cognition in schizophrenia and we discuss their relevance for putative behavioral interventions.

## Introduction

A recent integrative model (1), hypothesized a loss of synergistic interactions between cognition and reward circuits in schizophrenia. The model further argued that this loss underpinned deficits in learning and cognition (and many other generalized performance deficits) that are a hallmark of the illness (2, 3). Extant behavioral studies in patients have largely relied on reward-related contingencies (during reinforcement learning). Patients show deficits in reward anticipation, linked to reduced activity in regions of the mesolimbic pathway in the ventral striatum (4, 5). Patients also show reduced sensitivity in frontal-striatal circuits in experienced compared to expected outcomes (5, 6), and fail to faithfully represent the expected reward values of actions and outcomes (7). Generally, altered reward processing has been linked to intrinsic motivational deficits (3), where a loss of motivation in turn negatively impacts cognitive proficiency (8). A notable idea suggested by Robison and colleagues was that a loss of synergy between learning and reward circuits would be observed even if learning took place without explicit contingencies.

This link between cognition and reward processing is increasingly material to schizophrenia. A loss of motivation in patients has been associated with the perceived difficulty of tasks (particularly physical tasks) (9), and motivation loss drives patients to expend less cognitive effort to maximize reward. This reduction in cognitive effort further impairs task performance (10, 11), creating a maladaptive cycle. Finally, decreased cognitive effort appears to be inextricably linked with intrinsically a-motivational states (12).

Loss of synergy can be operationalized as a loss of functional brain network features (“connectivity”) (13–15). In the context of the current work, such loss would be observed between circuits underpinning cognition (16) and separate circuits in the mesolimbic pathway sub serving reward processing (17, 18). Thus, if altered reward processing and intrinsic motivational deficits impact cognitive proficiency (8), and if a hallmark of schizophrenia is a loss of both (3), then patients will show a loss of brain network features between cognitive and reward circuits even when learning without expectation of/or working toward reward.

Preliminary support for this idea comes from two sources: a) meta-analyses from a cross-section of studies (19) showing hypo-activation in the ventral striatum, and b) learning-induced dysconnectivity between regions like the ventral tegmental area (VTA) and the Nucleus Accumbens (NAcc), and other constituents of the learning network (15). These referenced studies need to be extended because meta-analyses have primarily included studies with explicit contingencies and the connectivity studies have not explicitly assessed the involvement of the VTA and the NAcc in any loss of connectivity. Here we addressed these lacunae as follows: 1) fMRI data were collected using a performance-driven associative learning paradigm without explicit reward or feedback (20, 21); 2) Next, from the fMRI time series data, 2^nd^ order network features (based on conventional zero-lag functional connectivity) (13, 22) were computed in each task condition for each participant across a common (SCZ ∩​ HC) network of bilateral nodes in the brain. The network included nodes from a learning-related circuit (including nodes in the cortex, the dorsal striatum and medial temporal lobe) (23–28), and nodes in the reward processing circuit (including the nucleus accumbens, NAcc, and the ventral tegmental area, VTA); 3) Then, within each group (SCZ or HC), higher order network features were estimated (see Methods). These features capture the consistency in connectivity between pairs of brain regions and their computation was inspired by “higher order functional connectivity” approaches designed to capture inter-regional resemblance of functional connectivity topographical profiles (29). 4) Finally, as an analogue to graph theoretic motivations (30), we quantified the relative contributions of nodes to the aberrant loss (or gain) of higher order features. Such quantification provides insights on the relative contributions of the VTA and/or the NAcc to higher order feature loss.

If learning and reward interject (even without explicit contingencies), we would expect the heavy representation of the VTA and the NAcc in any higher order feature loss (31, 32). This evidence would support the hypothesized loss of synergy between cognition and reward circuits in schizophrenia (1).

## Materials and methods

### Participants

Wayne State University’s IRB approved all procedures. Participants (n = 78) provided informed consent and were compensated. HC participants (n = 33; Range:18- 50 years) were free of psychiatric or neurological conditions. Patients (SCZ; n = 45; Mean Range: 18-50) were identified through their treating physicians (L.H., A.A.), and diagnoses were confirmed by a research psychologist (U.R.) using DSM-V criteria for schizophrenia (33). **Table 1** provides a comprehensive overview of the participant demographics.

**Table 1:** Demographic information for patients and controls are provided.

	SCZ (n=45)	HC (n=33)
Age (Years)	30.5 (± 7.8)	28.6 (± 7.0)
FSIQ	86.3 (± 6.9)	101.3 (± 8.5)
SASS	28.6 (± 9.0)	38.9 (± 5.4)
GAS	65.96 (± 5.4)	88.21 (± 5.1)
PANSS +ve	12.75 (± 3.5)	7.58 (± 0.97)
PANSS -ve	12.81 (± 3.7)	7.88 (± 1.04)
Duration of Illness (Years)	8.74 (± 7.9)	

45 patients (10 females) and 33 healthy controls (9 females) gave consent to participate. All patients were stabilized with antipsychotics (and in addition 20% with anxiolytics and mood stabilizers, 11% with antidepressants). The groups did not differ in age (p>.10) but expectedly differed on FSIQ (t_76_ = 8.6, p<.001), the Social Adaptation and Self Evaluation (SASS) scale (t_76_ = 5.8, p<.001), the Global Assessment of Function Scale (GAS) (t_76_ = 18.4, p<.001), the PANSS +ve and PANSS –ve respectively (t_76_ = 8.2, p<.001; t_76_ = 7.4, p<.001).

### MRI acquisition

fMRI data (3T Siemens Verio scanner, 32-channel volume head coil) were acquired using a multiband gradient EPI sequence (TR = 3 s, TE = 2.46 ms, multiband factor = 3, FOV = 192 × 192 mm^2^, matrix = 96 × 96, 64 axial slices, resolution = 2 mm^3^). T_1_-weighted MRI images were collected for normalization and co-registration with the EPI scan (3D Magnetization Prepared Rapid Gradient Echo (MPRAGE) Sequence, TR = 2150 ms, TE = 3.5 ms, TI = 1100 ms, flip angle = 8 degrees, FOV = 256 × 256 × 160 mm^3^, 160 axial slices, resolution = 1 mm^3^).

### Task

**Figure 1:**
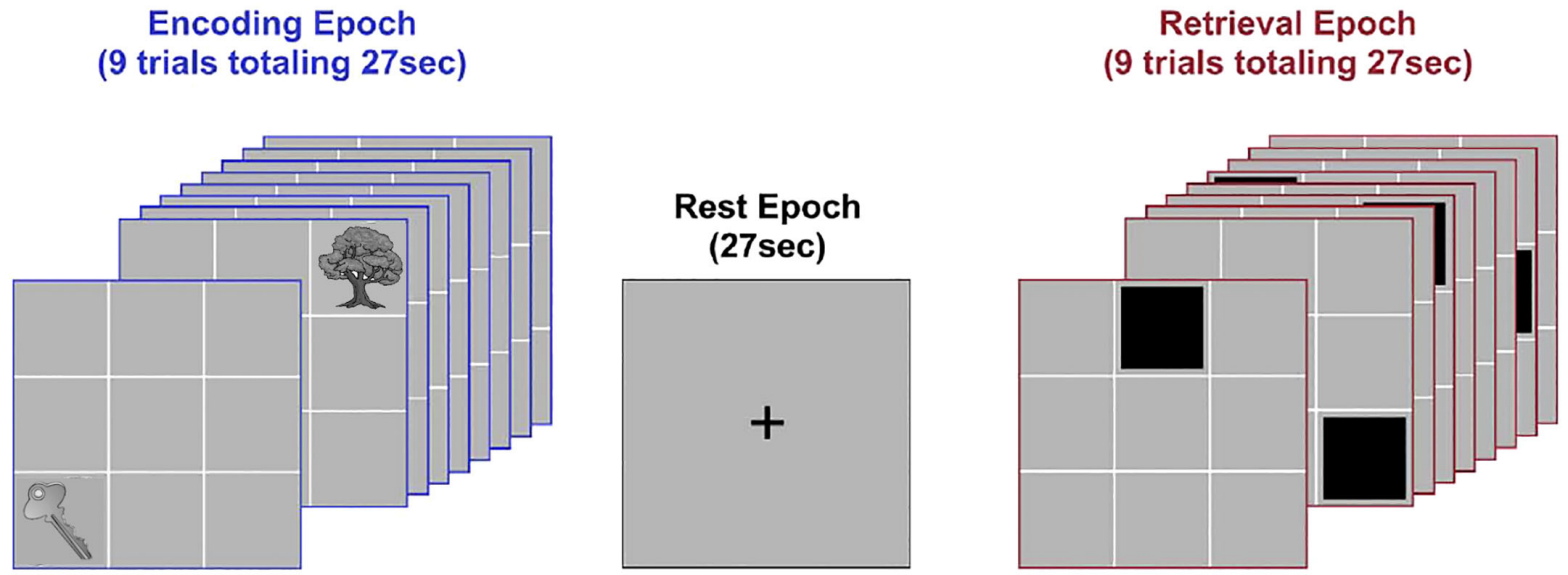
Task paradigm. The figure schematically depicts the deployed paradigm. Participants viewed illustrations of nine common objects presented in sequential random order in their associated locations during Encoding (3 s per object; 27 s total, successive presentations of “Key” and “Tree” are depicted). After a brief rest period (27 s), locations were cued in random order. Following each cue, participants were required to verbally name the associated object (Retrieval; 3 s per object; 27 s total). A brief post-retrieval consolidation period followed (27 s). The entire cycle went through eight iterations.

Network dynamics were evoked using an object-location associative learning paradigm (20). Subjects learned nine unique object-location associations (**Figure 1**), as the paradigm alternated between task-active epochs for Encoding and Retrieval (27 s each; ~13-minute acquisition)(with rest intervals interleaved). During encoding, nine equi-familiar objects (34) were presented in their associated grid location for naming (3 s/object). During retrieval (27 s), grid locations were cued (in random order) and subjects were required to name the associated object. Vocal responses were recorded by an experimenter using the pre-installed voice relay. During instruction-free rest intervals (27 s) participants were shown a cross-hair for fixation. Eight iterations of the learning cycle were employed. Participants received no feedback on their responses during Retrieval, and were not provided with any explicit contingencies (reward or punishment) for their performance.

### fMRI data processing and time series

fMRI data were processed in SPM 12 using standard methods for temporal (slice-time correction) and spatial preprocessing. For spatial pre-processing, EPI images were oriented to the AC-PC line, corrected head movement through realignment to a reference image in the sequence, and co-registered to the anatomical high resolution T_1_ image. The deformations from normalizing the high resolution T_1_ image were applied to the co-registered EPI images to normalize the volumes to stereotactic space. A low pass filter (128 s) was applied to remove low-frequency components. At the first level, epochs were modeled with boxcar stimulus functions convolved with a canonical hemodynamic response function to form regressors of interest. In each first level model, the six motion parameters (3 for translation and 3 for rotation) from the co-registration were modeled as covariates of no interest (analyses of the six displacement parameters indicated that estimated head movement did not differ between groups;.19 ≤ *p* ≤.91). Images were resliced (2 mm^3^) and a Gaussian filter (8 mm FWHM) applied. Images exceeding 4 mm of movement (<1% of all images) were excised from analyses.

First level maps were forwarded for a mixed second level random effects analysis of covariance. The singular goal of the second level model was to motivate the definition of a statistically reliable co-activated (across groups and task conditions) network. This co-activated network would form the basis for subsequent connectivity analysis, and the co-activated bases of the network would ensure that any inter-group connectivity differences are not confounded by inter-group activation differences (35, 36). In the second level model, Group (HC vs. SCZ) was modeled as independent factor, and Condition (Encoding, Retrieval) as non-independent factor, with covariates included for age, gender, and full scale IQ (FSIQ). We used Nichols et al.’s version of the minimum-inference statistic (37) (minimum statistic compared to the conjunction null) to identify an appropriate conjunction of co-activated clusters across groups and conditions. Statistical thresholding was based on cluster level inference (38) appropriate for both group and case studies (39, 40). A Monte Carlo alpha probability simulation was used to estimate the minimum cluster extent sufficient to reject false positive (noise-only) clusters. The simulation computes the probability of a random field of noise (after accounting for the spatial correlations of voxels based on the image smoothness within each region of interest estimated directly from the data set).

### Network space, time series and higher order feature analysis

The network definition was derived from a combination of meta-analytic priors patterns (41) and activation-based estimates paradigm (15, 21, 35). Priori studies have suggested that the learning sub-network includes portions of the early visual stream, including a) the occipital lobes (OCC) and the fusiform gyrus (FF), known to function in object identity processing (23–25, 42), b) the parahippocampal gyrus (PHG) and the body of the hippocampus (BHIPP) involved in episodic-based learning and memory (26, 28, 43), and c) frontal and striatal regions including the dorsolateral prefrontal cortex (DPFC), and the basal ganglia (BG) associated with task-relevant sub-processes for attention, working memory and contributions to the early stages of longer-term memory formation (44, 45). Notably, the basal ganglia also support working memory maintenance through frontal-striatal loops (46–48), and play a role in reward-prediction (49–51). Anatomical masks based on these regions were used as a filtration for the conjunction analyses (see below).

This learning circuit was complemented by nodes representing the NAcc and the VTA. The NAcc appears to direct behavior toward rewarding, or away from aversive stimuli (31, 32, 52, 53). More specifically, NAcc’s contributions toward goal-directed behavior are amplified in the context of external reward-based contingencies (54), where the explicit contingencies appear to prime the interjection between reward and cognition. Thus as a crucial constituent of the dopamine reward circuit, the NAcc is an interface between motivation and action (55). The structure receives neuronal projections from the VTA, and the NAcc and the VTA are the principal drivers of reward related contributions of the mesolimbic pathway toward goal-directed behavior (56–58). The mesolimbic dopaminergic system thus plays a central role in motivated behaviors (59), particularly in scenarios involving in external rewards (e.g., monetary incentives) (60). For the learning network, individual significant peaks were identified from within anatomical masks under regions of interests in a deterministic anatomical atlas (61).

**Supplementary Figure 1A** depicts the results of the conjunction analyses and the harvested loci in each of our regions (with labels), are depicted in **Supplementary Figure 1B**. All subsequent connectivity analyses were based on time series (averaged first eigenvariate) extracted from the depicted nodes (4 mm radius). The experimentally derived coordinates (depicted in red) were complemented by the meta-analytically derived coordinates for the NAcc and the VTA (depicted in grayscale) (41). For each of the NAcc and the VTA, an experimentally derived search space (10 mm diameter) was centered around priors (41), before identifying peaks for each participant within this space.

**Figure 2:**
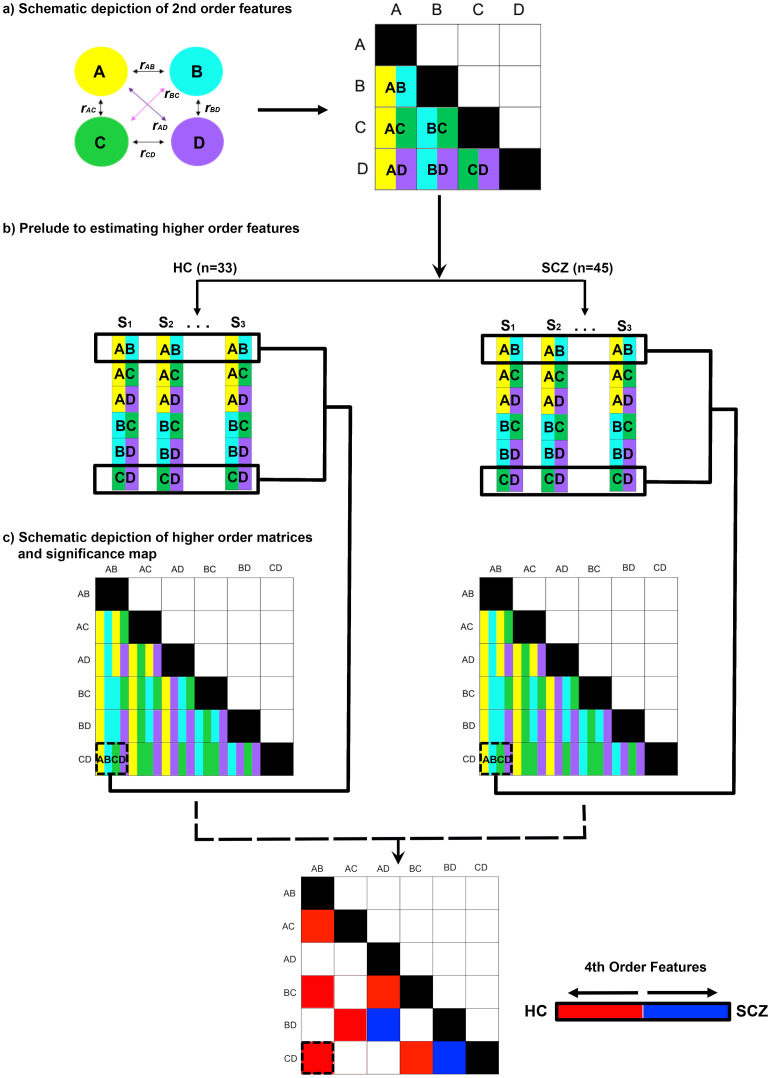
Methods outlined. The pipeline used to estimate higher order features is depicted. **(A)** In an initial step, the full second order functional connectivity matrix was formed for all sixteen nodes in the network (schematically depicted for four nodes, A-D). The matrix was derived from time series for each node, with each participant contributing matrices for Encoding and Retrieval. The undirected functional connectivity (uFC) was computed as the zero-lag correlation (*r)* between all unique pairs of nodes (i.e., *r_AB_*) for each subject, with the coefficient normalized (see Methods). Each correlation measure represents by convention a 2^nd^ order connectivity feature. **(B)** Across all participants in each of the HC and SCZ groups, higher order features were estimated from these 2^nd^ order matrices. As depicted for *r*_AB_ and *r*_CD_, in each of the HC and SCZ groups, the resultant higher order feature (*r*_AB_ • *r*_CD_) was estimated as the correlation in 2^nd^ order features across all 32 and 46 participants respectively. **(C)** The resultant higher order feature matrices represent the intra-group regularities across quadruples of network nodes. From these, intra-group differences in regularity were estimated (see Methods) and statistically thresholded (bottom).

The extracted time series were forwarded for undirected functional connectivity (uFC) analysis (using bivariate zero-lag correlations) (13). Temporal vectors from the task paradigm were used to separate the time series into each of the task-active conditions of interest (Encoding and Retrieval). Then, for each subject, the full uFC adjacency matrix [120 unique node pairs, *C*(_16,2_)] was estimated in each task condition (implemented with in-house scripts in Matlab) before the Pearson’s *r* values were transformed using the Fisher’s *Z* transformation (62). Each cell value represents conventional 2^nd^ order network features between pairs of nodes (e.g., for nodes A and B, *uFC*_AB_) in any given participant. **Figure 2A** schematically represents this part of the analytic pipeline (for a hypothetical four-node network space).

These 2^nd^ order features formed the primary data for estimating higher order features (**Figure 2B**). Here, within each group, the cross-correlation computed between every possible pair of second order features (e.g., *uFC*_AB_ × *uFC*_CD_), provides an index of the statistical regularity between that pair of 2^nd^ order features. This estimate is an analogue of measures of the macroscopic consistency between pairs of brain circuits (63). In each group and in each condition, we constructed a higher order feature matrix from all possible combinations of 120 unique 2^nd^ order features. This higher order feature matrix consisted of 7140 unique cells [*C*(_120,2_)]. The values in each cell were transformed and normalized, *r’* (64), to estimate inter-group differences in *r’* (SCZ ≠ HC) using the *z* statistic (65). The *z* statistics were thresholded (*q*_FDR_<.05) (66) to identify significant inter-group differences in the consistency of higher order features across the matrix (see **Figure 2C**).

## Results

**Figure 3:**
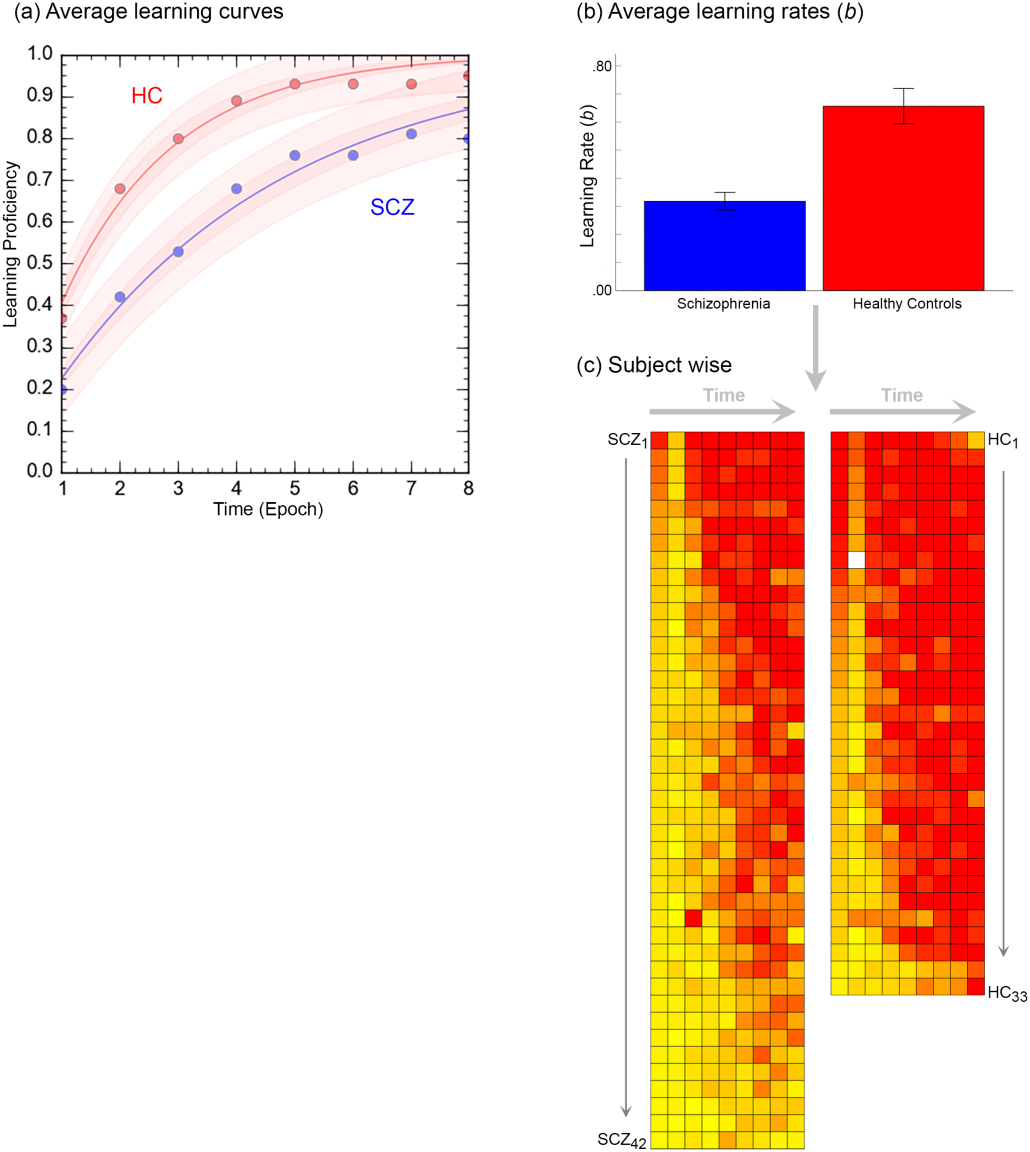
The figure provides a comprehensive accounting of the behavioral results. **(A)** Averaged learning data at each time point are presented where the curves represent negatively accelerated learning functions, $y=1-e^{-bx}$ , fit to the averaged data (shaded areas represent 95% confidence intervals). As seen, patients display slower learning. This intuition was confirmed in **(B)**. Here, learning functions were separately fit to performance data from each of the participants, and the average learning rates, *b* are depicted in the bar graph (± s.e.m.). The bar graph confirms that learning rates in patients was significantly lower. **(C)** Finally, individual performance data were rendered using heat maps. Here each row depicts data from an individual participant (each column represents time, i.e. epoch). Participants in each group are arranged in descending order of learning rate (Top: Fastest → Bottom: Slowest).

**Figure 4:**
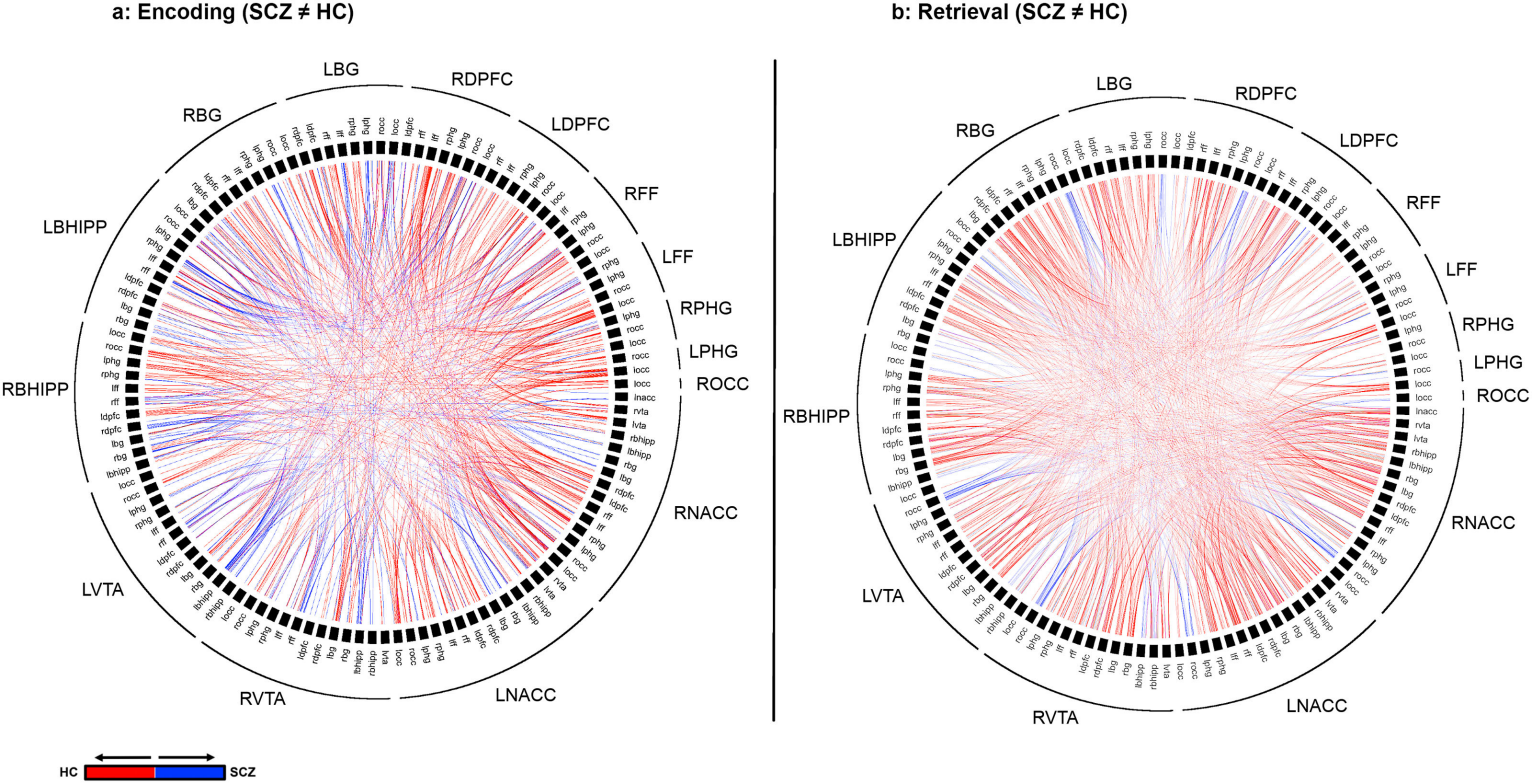
Differences in higher Order Network Features for each of Encoding **(A)** and Retrieval **(B)** are represented using chord diagrams. To denote the specific higher order feature, the 16 node identities are denoted on the outer ring. Then, the labels in the inner ring are organized to form a unique pair of nodes that form one of the elements of the higher order feature. Finally, each visible chord links pairs of network pairs denoted by the combination of the outer and inner label. Red chords connect pairs with greater higher order significance in in HC while blue chords connect pairs with greater higher order significance in SCZ. As seen, SCZ are characterized by a massive loss of higher order network features.

**Figure 5:**
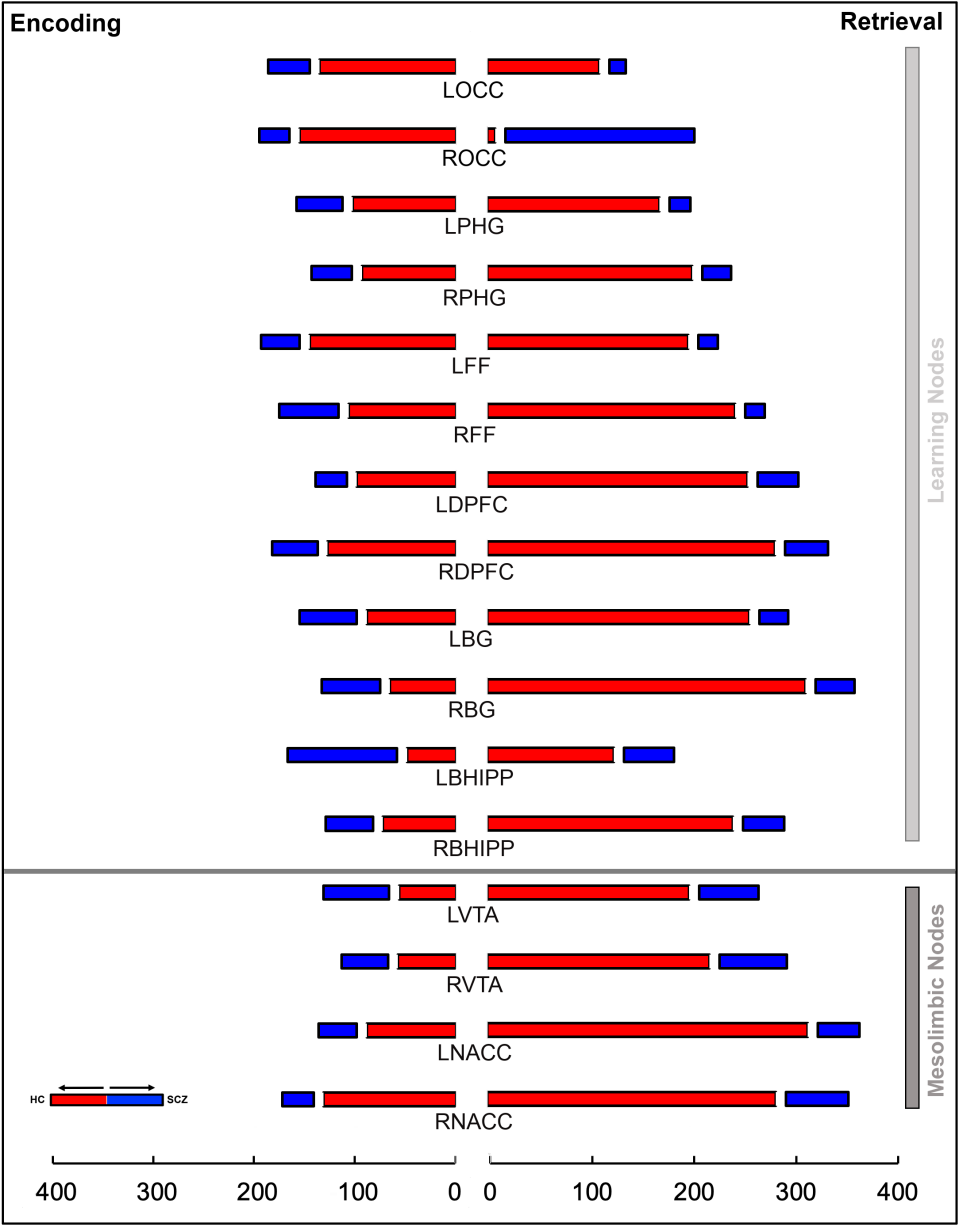
The stacked bar graphs represent the total number of instances a node is a member of a given chord in **Figure 4**. These graphs are constructed separately for Encoding (left) and Retrieval (right) conditions. The graphs provide a measure of the relative contribution of any node to a loss or gain of higher order features in schizophrenia in each of the conditions. The color scheme (red/blue) is maintained from **Figure 4**. In addition to the notable contributions of regions central to learning and memory, the contributions of nodes in the mesolimbic system (bottom), particularly during Retrieval, are highly salient.

**Figure 6:**
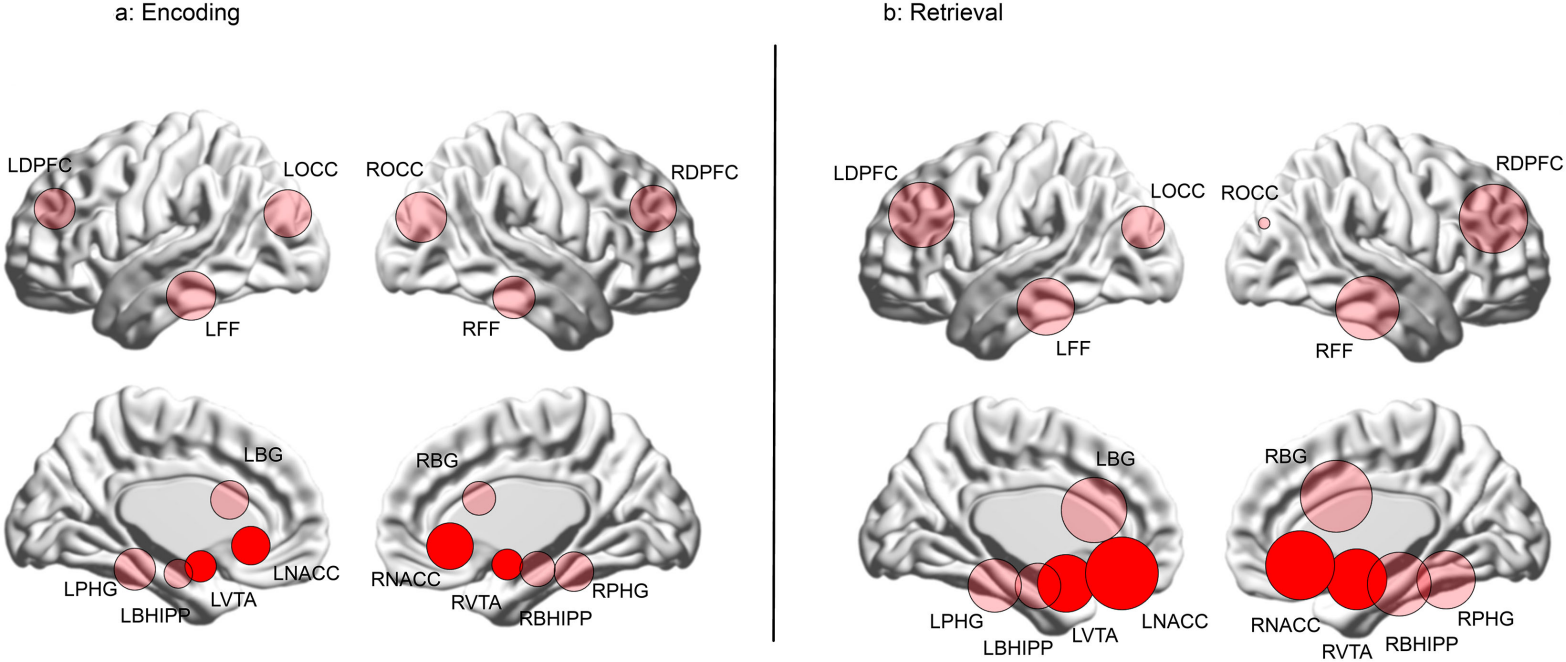
The data from **Figure 5** are re-visualized for each of the **(A)** Encoding and **(B)** Retrieval conditions as numerically scaled circles (only where there is a loss of higher order features) where each circle represents a node placed in its approximate anatomical location on a lateral or medial surface of the brain. Each node’s diameter is scaled to reflect its contribution to higher order feature loss: diameter $d=\frac{\sqrt{Chord}\;Frequency}{2\;x\;10}$ . The relative contributions of the nodes in the mesolimbic pathway (NAcc and VTA) are highly evident by their depicted sizes (highlighted for reference).

The results are organized as follows: a) We first provide the behavioral data from the task (**Figure 3**); b) Next, inter-group differences in higher order group features are presented using connectomic rings (**Figure 4**). The chords in each ring (Encoding and Retrieval respectively), denote significant inter-group differences in higher order group features (see **Figure 2C**, bottom). Each chord links two pairs of region pairs (formed by the region names on the outer ring and the inner ring); c) **Figure 4** provides a gestalt of the results, but is not informative about the relative contributions of each node to the observed inter-group differences. In other words, how implicated is any node in a loss of higher order features? To capture this information, data from **Figure 4** are re-expressed in **Figure 5**. Here, the frequency graphs indicate the frequency with which each node was present in any significant inter-group difference (i.e., present in each chord in **Figure 4**). These observed frequencies were subsequently submitted to non-parametric statistical analyses (X*^2^*) (67); (d) Finally, the deficit data from **Figure 5** (SCZ < HC, red bars) are summarized on a brain map (**Figure 6**). Here, the size of each anatomically placed node is scaled by that node’s observed frequency (**Figure 5**). Thus, **Figure 6** motivates the direct visual assessment of the importance of any single node to higher order feature loss in SCZ.

### Behavioral results

**Figure 3** provides an accounting of the behavioral results (data from three patients was lost on account of errors in recording responses over the voice relay). Behavioral results (proportion correct during each retrieval epoch) were analyzed in a two-way repeated measures analyses of covariance with factors for time (modeled as the repeated factor) and group (modeled as the non-repeated factor; age and FSIQ were modeled as covariates). Two salient effects emerged. First, we observed a significant main effect of time, *F*_7,497 =_ 2.68, *p*<.01, *MSe*=.213, indicating that performance improved over time. Second, we observed a main effect of group, *F*_1,71 =_ 11.16, *p*<.001, *MSe*=.363, indicating that learning proficiency was impaired in patients. Averaged learning curves are presented in **Figure 3A**.

Next, negatively accelerated learning functions using the classic single parameter psychometric learning function, $y=1-e^{-bx}$ ,were fit to available data from each of the 75 participants. Here, the parameter *b* represents learning rate (higher values indicate faster learning and faster transitions from linear to asymptotic performance) (68–70). The bar graph in **Figure 3B**, indicates that learning rate in patients was significantly lower, *t*_73 =_ 5.12, *p*<.001, confirming the visual impressions from the data in **Figure 3A**. Finally, in **Figure 3C**, performance data in individual participants are rendered using heat maps. Here each row depicts data from an individual participant (each column represents time, i.e. epoch), with participants in each group arranged in descending order of learning rate.

### Inter-group differences in higher order features: encoding & retrieval

**Figure 4** depicts significant inter-group differences in higher order features during Encoding (a) and Retrieval (b). The colors represent the direction of feature loss (Blue: SCZ_(A←→B, C←→D)_ > HC_(A←→B, C←→D)_; Red: HC_(A←→B, C←→D)_ > SCZ_(A←→B, C←→D)_). Each chord represents a result involving two 2^nd^ order features. Therefore, the identities of the pairs can be derived from the combination on the label on the outer ring and the label on the inner ring.

The overall gestalt indicates two salient effects: a) During *both* Encoding and Retrieval, higher order feature loss was extensive in patients, with effects particularly pronounced during Retrieval. Next, we attempted to motivate inference on the contributions of individual nodes to this effect. Thus, for each of Encoding and Retrieval, we first calculated the frequency of every node’s involvement in aberrantly lost (or gained) second order features. These data are expressed in the stacked frequency bar graphs in **Figure 5**. The width of the bars express the frequency profiles for each node. This in turn, permits visual inspection of their relative importance in feature loss (or gain) *vis a vis* other nodes in the network.

During Encoding, nodes like the bilateral occipital cortex, the right dPFC, the left fusiform cortex, and notably the right NAcc contribute heavily to higher order feature loss in SCZ. The relative contributions of nodes in the mesolimbic pathway is amplified during Retrieval. Here, each of the bilateral nodes in the VTA and the NAcc, and right Basal Ganglia contribute heavily.

Next, we tested whether the observed frequencies of the contributions to the *loss* or *gain* of features was different than the expected frequencies (under the null hypothesis that these frequencies would be evenly distributed). This analyses was separately conducted in each condition using a two-way (Node and Group) X*^2^* analysis using the X*^2^* goodness of fit test:


$$X^{2}=\sum \frac{{\left(observed-expected\right)}^{2}}{expected}$$

We observed significant effects for *both* Encoding and Retrieval: Encoding: X*^2^=* 205.59, df=15, *p*<10^-5^; Retrieval: X*^2^ =* 908.08, df=15, *p*<10^-5^, indicating that the nodal contributions to higher order feature loss was unequal, and that mesolimbic nodes like the VTA and the NAcc contributed substantially, particularly during Retrieval.

### Visualizing the relative contributions of nodes

The relative contributions of each node to the loss of higher order group are visualized in **Figure 6**. Here, each node is placed in its approximate anatomical location (either on a lateral or medial cortical surface), with node size scaled as a function of its frequency in **Figure 4** (diameter $d=\frac{\sqrt{Node}\;Frequency}{2\;x\;10}$ *)*. Nodes in the mesolimbic pathway (VTA and NAcc) are shaded in darker red. As seen, during Encoding (**Figure 6A**), nodes associated with vision and object recognition, including occipital (ROCC and LOCC) and fusiform nodes (RFF and LFF) contribute heavily to higher order feature loss, as do nodes in the frontal cortex and the basal ganglia. Finally, the NAcc contributes substantially, underlining its relevance for learning deficits in schizophrenia (31, 32). The relative contributions of the bilateral NAcc and the VTA are amplified during Retrieval (**Figure 6B**).

## Discussion

Our principal goals were to investigate whether learning without contingencies (or feedback) induced a loss of higher order network features in schizophrenia, and to assess the relative contributions of the mesolimbic system to this loss. Any higher order feature loss (or gain) was characterized during two task-active epochs (Encoding and Retrieval). Our results revealed: 1) that patients showed a cumulative loss of higher order features during both task-active conditions (**Figure 4**); 2) The contributions of the nucleus accumbens and the ventral tegmental area equaled or exceeded most other nodes in the investigated network (**Figures 5**, **6**).

If a loss of synergy between cognition and reward circuits/regions is central to schizophrenia (1, 71), and if this loss is evoked independent of external contingencies, then one would expect heavy representation of regions like the NAcc and the VTA in our results (as indeed we observed). The remainder of the paper discusses aspects of the network bases of learning, the interpretation of higher order feature loss, and the plausible clinical relevance of these findings.

### Network bases of learning

Learning is a dynamic, complex process featuring multiple interacting brain regions that form feedback and lateral loops (72). Our task loosely conformed to two general processes/stages linked with learning, specifically (1) Encoding, a dynamic process of perceiving new information or memoranda, initiated into the early process of memory formation and (2) Retrieval, the ability to access and recapture stored memory traces on demand (73–75). These processes involve regions including the Hippocampus, Para-hippocampus, the Basal ganglia and the DPFC (76), as well as other posterior brain regions associated with visually-driven learning (77). The roles of the NAcc and VTA on the other hand have primarily been studied in the context of motivational learning (78) and interactions between these regions and the hippocampus are assumed to be important in tuning the sensitivity to novelty. Sensitivity to novelty is a crucial element in learning and long-term memory formation (79, 80). The VTA strengthens encoding and retrieval by facilitating hippocampal and prefrontal co-activity (81, 82), and such mediation is presumably sub served by its dopaminergic projections. Thus, the encoding of novel information engages the hippocampus (83–85), thereby aiding the generation of memorial representations. These representations are in turn employed by the prefrontal cortex during executive functioning and working memory (86). In addition, cohesive activity between the basal ganglia and the hippocampus underpin the learning of arbitrary associations (87). Finally, the DPFC in contributing to multiple stages of learning, promotes the cognitive processes necessary for behavioral planning, executive processing, top-down processing, active maintenance of stored memories and cued retrieval (45, 88–91). This distributed narrative is consistent with the multiple memory systems theory (MMS) which postulates that information is stored based on the independent and parallel activity of a multiple brain modules, where each has distinct properties, dynamics, and neural bases (92).

The link between reward processing and cognitive behavior is central in the animal literature; the reward system sends modulatory inputs to regions like the hippocampus and entorhinal cortex, and these inputs are essential to behavior (93). However, animal studies (largely conducted at the cellular level) and human neuroimaging studies (almost entirely conducted at the system’s level) cannot easily be reconciled. Cellular processes are not straightforwardly expressed in neuroimaging signals (94) and human network architectures for any task can coopt a greater array of resources and rely on a greater degree of network complexity (95–97). These challenges notwithstanding, we draw tentative inferences regarding the clinical relevance of the observed higher order feature loss in schizophrenia.

### Interpreting higher order feature loss in schizophrenia

As defined in our analyses, higher order features index the statistical relationship between any two second order features, where each second order feature is itself a relationship between two variables (98). The data in each participant represents a summary of a multi-variable complex system (e.g., a brain network) (14) and across members within any group (e.g., patients or controls), a higher order feature provides a measure of the intra-group consistency between pairs of system elements. Therefore, these features integrate across measures of functional connectivity in groups of participants (13). In the context of macroscopic network neuroscience (99), such higher order measures may aid in characterizing intra-group network organization, or (as we have done), or aid in identifying inter-group differences, and the relative contributions of nodes to these differences. The latter is somewhat analogous to the use of graph theory, which is frequently used to summarize nodal importance in brain networks (100, 101). From **Figures 5**, **6** (which elucidate the role of individual nodes in higher order feature loss), we can draw inferences on the importance of each to learning-related impairments in schizophrenia.

The representation of bilateral occipital nodes during Encoding (**Figure 6A**) underlines the integrative role of visual processing in learning and cognition (102) suggesting that learning-related network dysfunction in schizophrenia may originate in the primary and secondary (magno- and parvocellular pathways) visual pathways (42, 103). The representation of bilateral fusiform nodes reaffirms the important role of the forward visual pathways in schizophrenia where deficits in anatomy and function have been widely reported (104, 105). The representations of the DPFC and the BG are interpretable based on their central roles in memory, attention, and executive function (106, 107). Finally parahippocampal contributions to feature loss are consistent with the structure’s role in the early stages of memory consolidation (26, 28, 43).

In this context, the significant representation of both the NAcc and the VTA in is particularly salient. As noted, the NAcc plays a key role in tasks where learning is yoked to contingencies or external feedback (31, 32, 52, 53) but its role in the absence of feedback or external reward is less understood. This latter role may well loop into latent processes associated with internal motivation or motivated behavior (108). In the absence of explicit reward, the NAcc and the VTA may autonomously integrate different sources based on the assessment of implicit value (109). Moreover, the VTA’s heavy representation is also consistent with its role in linking motivation and action (110), where this link may be compromised in schizophrenia (111, 112). More generally the mesolimbic system plays a well-established role in facilitating reinforcement and motor learning through motivation and appetitive desire (56–58), but this role appears to be substantially impaired in schizophrenia patients during encoding even in the absence of reward related contingencies.

Retrieval, the only performance driven phase of the task amplified feature loss. During memory retrieval, cues to facilitate access to internally distributed memory representations originate in the prefrontal cortex (45, 88, 113) but may be compromised in schizophrenia for multiple reasons: a) functional deficits in the medial temporal lobe undermine memory formation (114), resulting in b) poorly consolidated associations that undermine subsequent retrieval (115) that are further compromised by hypo-functionality of the prefrontal cortex (116, 117). While prior accounts have suggested that hallucinatory experiences (by occupying functional space in the medial temporal lobe) interfere with the process of episodic memory formation (118), our results imply that the contributions from impaired functions of the mesolimbic system may be equally salient. Retrieval appears to rely on integration of the NAcc and the VTA in network-based memory signaling (119), because these signals also transmit information about the interjection between the affective states (elicited by successful recall) and cognitive processes (120) that are enhanced under positive affect (121). Therefore, a disease process like schizophrenia will be associated with the reduced integration of the NAcc and the VTA with cognitive circuits (1).

Data from at least one clinical scale provides a measure of clinical relevance to our results. The social adaption and self-evaluation scale (SASS) (122) is a 21-item scale developed to quantify social motivation and behavior. Studies have confirmed its validity in assessing perspective on self, environment perception, and social motivation in both healthy and clinical populations (123–125). Lower scores are indicative of a loss of motivational and hedonic drive, and this loss has been linked to altered processing of reward-related salience and diminished responses of the NAcc and the VTA (126, 127). Schizophrenia patients score significantly lower on the SASS (128), and this evidence validates the clinical impression that patients suffer from a loss of intrinsic motivation, curiosity and hedonic drive (129). Results from our sample are consistent with cited evidence. Patients scored significantly lower on the SASS (**Table 1**) suggesting a loss of intrinsic motivation, and (expectedly), patients also evinced significantly greater negative symptoms (PANSS). We cannot specifically relate deficits in the SASS with higher order group features, because by definition, the latter are defined at the group level (see **Figure 2**) and do not exist for an individual participant. However, in ongoing work (130) we are using complementary methods to partially addresses this shortcoming. Each participant’s *N* region 2^nd^ order functional connectivity (FC) matrix is transformed into an *N* region higher order functional connectivity matrix (HOFC) (131). Each cell in the HOFC matrix encodes the inter-regional resemblance of the FC topographical profiles and can therefore be related to clinical measures. However, HOFC is distinct from the network features defined in the current investigation and further analyses will be need to elucidate the degree to which the two are complementary or supplementary.

### Limitations and conclusions

Reverse inference is challenging (132, 133) because imaging results under-specify clinical relevance. However by modus tollens (If A, then B. ~B, therefore ~A) we can derive logically sound inferences regarding network dysfunction in neuropsychiatric illness (14). Accordingly, if the brain is intact, then we expect a certain pattern of network properties; If we do not find that pattern of network properties, then the brain is not intact. The challenges of reverse inference are acute for resting state analyses (134), but they are somewhat mitigated in the context of tightly defined experimental tasks. If tasks are reasonably well mapped to brain networks, then task conditions can also be reliably mapped to those network constituents (135). Accordingly, our choice of task and our analytic method were purposefully synchronized to a) examine the impact of learning without contingencies on b) contributions of the mesolimbic system to the loss of higher order structure in schizophrenia, before c) quantify the role of each of the NAcc and the VTA in b). With that said, the import of our work is constrained by notable limitations.

First, higher order features do not have any specific neuronal correlates, nor can any such correlates be derived because as examined here, these features are only defined at the level of the group (see **Figure 2**). This distinguishes our approach from studies that use functional (or effective) connectivity (13, 22); these methods use statistical (or generative) models that characterize “connectivity” at the level of the participant. Thus, higher order features are not a new *model* of network connectivity; rather they allow one to efficiently summarize the consistency of network connectivity (defined in each participant) across all participants in an *a priori* defined group. Conventional statistics can then be used to highlight inter-group differences in this consistency. In principle, this approach can be applied to any class of estimated 2^nd^ order features, and we suggest that its neuronal relevance will be better understood when applied to electrophysiological data.

Our work would ideally want to parse apart effects associated with successful versus unsuccessful recall (136). Because successful recall appears to obligatorily engage regions in the ventral striatum (137), it may be intrinsically more rewarding than recall failure. However any such analyses would at the very least demand an event-related design, using which one could post-hoc cleave apart correct versus incorrect recall trials (138–140). However, here we were compelled to use a block design (without jittering of inter-stimulus intervals) (141) because recall was assessed by requiring participants to name the objects associated with the cued location (see Methods). Accordingly, each stimulus presentation was locked to each TR (and the whole brain image was acquired within the TR window, with a period of silence within which participants responded). Moreover, we were not merely interested in activation differences between recall success and recall failure (142). Rather, our analyses were predicated on the ability to first estimate functional connectivity from time series data, a class of analyses not typically used event-related designs, because the latter are optimized to identify differences in activation to difference classes of events. Nevertheless, and as noted earlier, published evidence using event-related designs supports some of our findings. Retrieval success (as opposed to failure) is associated with activation of the ventral striatum (137, 143), supporting our analyses where at a more macroscopic scale we captured effects associated with the actual task conditions (**Figures 4**–**6**).

Finally, we do not have a model for specifically how the NAcc and the VTA are involved when learning without contingencies, though widespread evidence indicates that regions like the NAcc play highly a generalized role relating to reward and threat. For instance, human studies have shown that when correct responses on a social incentive task are “rewarded” with simply the presentation of a positive expression from a romantic partner versus a stranger, activity patterns in the NAcc are strongly discriminative between the former and latter (144). Such studies imply that the NAcc may be involved in selective preference or salience. Thus, it is plausible that our observed results represent the inability of schizophrenia patients to experience the salience of reward (145). Further evidence of the broad scope of NAcc function comes from recent animal work. Rather than simply being involved in responses to reward, recent work shows that populations of NAcc neurons alter their firing patterns to cues indicating threat (146). Such results reinforce the crucial role of the mesolimbic system in supporting and organizing goal oriented appetitive and avoidant behaviors, and it is precisely this synergy that is lost in schizophrenia (1, 15).

## Data Availability

The original contributions presented in the study are included in the article/**Supplementary Material**. Further inquiries can be directed to the corresponding author.
