# Supplementary figures and images for: The mesolimbic system and the loss of higher order network features in schizophrenia when learning without reward
Source: Front Psychiatry. 2024 Sep 3;15:1337882. doi: 10.3389/fpsyt.2024.1337882 (PMC11443173; doi:10.3389/fpsyt.2024.1337882)

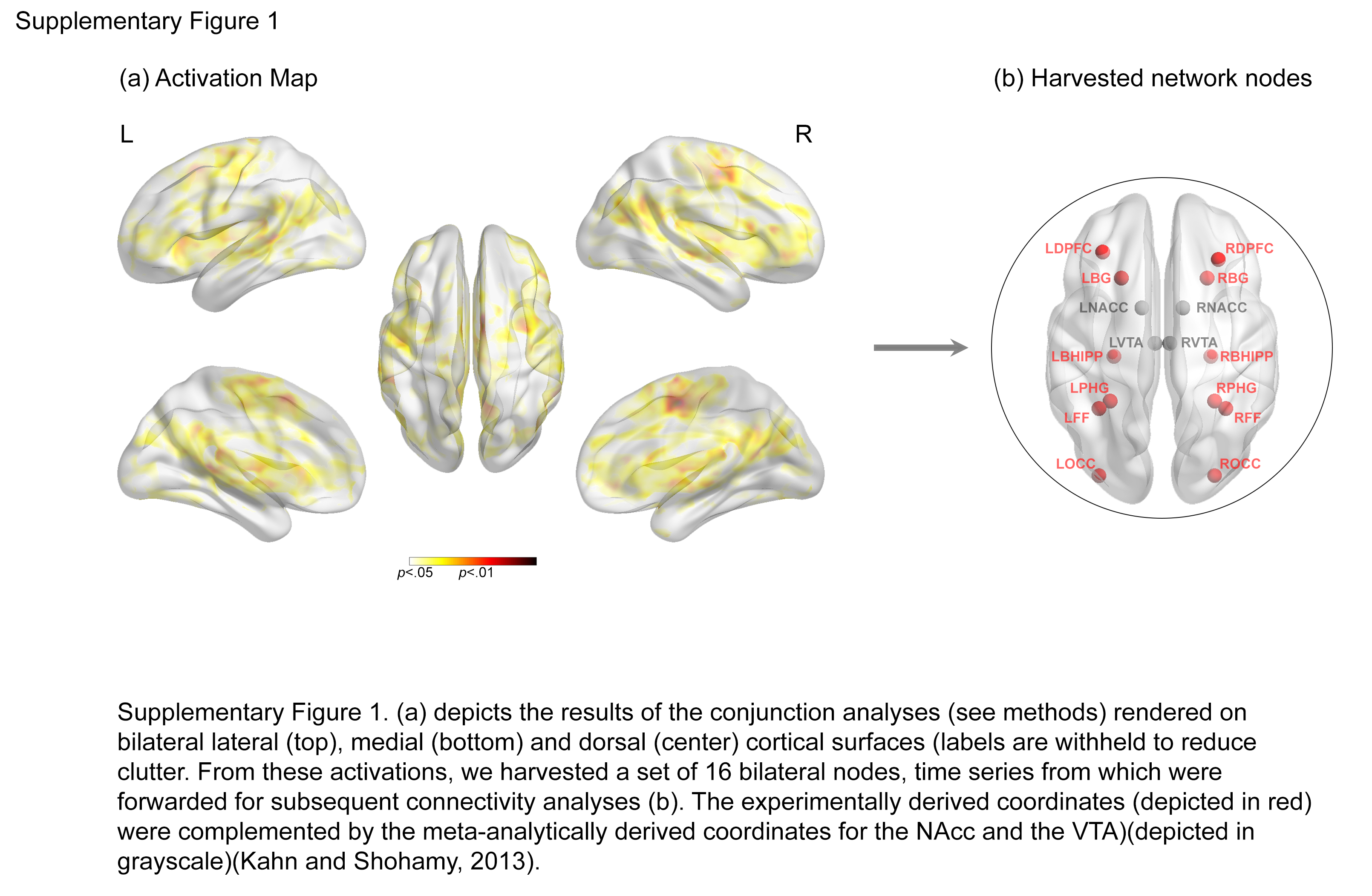

Supplement: Supplementary file 1 [file Image1.tif]
